# We Need Our Village: CORD’s Response to the ACGME’s Common Program Requirements

**DOI:** 10.5811/westjem.2019.7.44138

**Published:** 2019-07-03

**Authors:** Maria E. Moreira, Christopher I. Doty, Fiona E. Gallahue

**Affiliations:** * Denver Health and Hospital Authority, Department of Emergency Medicine, Denver, Colorado; † University of Colorado School of Medicine, Department of Emergency Medicine, Aurora, Colorado; ‡ University of Kentucky-Chandler Medical Center, Department of Emergency Medicine, Lexington, Kentucky; § The University of Washington, Harborview Medical Center, Department of Emergency Medicine, Seattle, Washington

“It takes a village to raise a child,” is a common colloquialism referring to the immense amount of time and support required to develop a contributing member of society. In residency education, it takes collaboration amongst multiple individuals to run a program, educate within the standard of graded responsibility, and mentor residents with differing needs and interests. This village of educators (Residency Leadership and Core Faculty), allows for exposure to different ways of thinking, approaches to patient care, and methods of teaching, creating a rich environment for the exponential growth of learners. It is the responsibility of these individuals to assure excellence in education, inside and outside the clinical setting, to develop high quality emergency physician (EP) graduates for our society.

Until 2019, the Emergency Medicine ACGME (Accreditation Council for Graduate Medical Education) program requirements stated that institutions were required to provide protected non-clinical time for core faculty. Specifically, core faculty could not be required to generate clinical or other income to support that protection. These core faculty could not average more than 28 clinical hours per week, or 1344 clinical hours per year. In the new proposed program requirements, the requirement to ensure this non-clinical time has been removed. This will undoubtedly have a negative effect on the quality of resident education and the physician wellness of the faculty.

While we can certainly understand the ACGME’s desire to create uniformity in processes among training in all specialties, there are unique qualities of each specialty that merit individualization. Specifically, EM has shift scheduling challenges, increasing patient volumes with frequent emergency department (ED) crowding, and an increased burden of clerical work. These factors pose unique challenges in educating and training EM residents that will create serious consequences without the provision for protected time for clinician-educators. Additionally, changes in other specialties and decreased availability of specialists has led to increased workload on EPs, and increased need for education in areas that were previously not in the purview of our specialty.

The ED is open and available 168 hours per week, all weekends and all holidays. An EP’s work is compounded when other specialists are less or not available at all. It has been demonstrated that when EDs are busy, EPs need to be able to distribute the work of procedures to admitting services to continue to serve the patients.^1^ Current ED trends demonstrate increasing volumes and complexity each year, which further challenge EM educators to teach during clinical shifts.^2^

Several changes in other specialty requirements have been focused on controlling the learning environment to comply with ACGME rules. These, have, in turn, negatively impacted EM residents by increasing workload. Patient capping (limits for inpatient residents to accept further patients for care), decrease in non-EM specialty procedural requirements (creating need for EM residents to perform procedures prior to admission), and changes in rotational requirements (removing off-service residents from ED rotations) have all impacted the ED. The effect of capping patient volumes to admitting services has increased ED crowding, which increases the cognitive load on EPs.^3^ When other specialties decrease their scope of practice, the EP’s must necessarily expand, increasing the workload and complexity of the learning environment. As an example, the removal from the ACGME requirement for nephrology fellows to be trained to place dialysis catheters has shifted that responsibility to critical care or ED teams. With the decrease in other specialties rotating through the ED, the understanding of the ED environment by consultants and admitting teams declines as well. This lack of exposure and understanding can lead residents to delay writing admitting orders until it is either convenient, or the patient has been seen by more senior residents and supervisors, or even other possible admitting services. This leads to delay in patient care, increased cognitive load on the EP, and patient dissatisfaction. Additional stress may decrease EP empathy, the faculty’s ability to educate, and residents’ openness to learning.^4^

The scope of EM practice is very broad. One of the critical aspects of EM training is preparing learners for low frequency, high stakes clinical scenarios and procedures. As the scope of practice for procedures done in the ED continues to expand, the burden of education that occurs outside the clinical environment increases. Peri-mortem c-sections, emergent cricothyrotomies, acute resuscitations of massive gastrointestinal bleeds, and ruptured ectopic pregnancies are not very common scenarios, but an excellent EM resident must be prepared and competent to perform these rare clinical cases. What allows training residents to achieve competence is the increased use of high and low fidelity simulators and task trainers. Proper preparation of learners for these cases requires innovative teaching strategies that leverage technology, simulation, blended learning, and traditional teaching. To guarantee exposure of all residents, procedural experiences and other teaching must be scheduled outside of the ED clinical environment. EM education “beyond the shift” has been identified as a best practice, given common ED crowding, which limits time available to teach due to immediate patient care needs.^5^ Suggestions include that faculty send articles after shifts and create teaching files outside the shifts to best educate EM residents. For procedural training, simulation is increasingly necessary to ensure patient safety and a standardized training environment. The number of procedures done and self-report of comfort does not equate to procedural competence.^6^ “Rigorous simulation-based education is a natural fit with the ACGME milestone framework because it provides standardization, deliberate practice, feedback, translation of outcomes to improved patient care, and reliable formative evaluation until a mastery standard is met.”^6^

While EM faculty are committed to providing these blended teaching methods and experiential learning environments, they require protected non-clinical time for preparation and teaching. Such examples from EM educational faculty in ultrasound and simulation demonstrate the time commitment of these training modalities outside of the clinical environment.

- Benchmarking surveys performed by The Society for Clinical Ultrasound Fellowships determined that clinical ultrasound faculty spend, on average, 590 hours per year on ultrasound activities, with 288 hours spent on ultrasound education alone. This translates to more than 6 hours per week per faculty member. An additional 124 hours per year is spent on quality assurance of ultrasound examinations performed by residents, fellows, and faculty as part of the education mission.
- Data from the Society for Academic Emergency Medicine’s Simulation Academy demonstrates that, on average, 300 hours of simulation are taught every year to students, EM residents, and fellows by each EM simulation faculty. This survey also demonstrated that most programs are using simulation to educate EM residents with up to 30% of curriculum being taught via simulation and faculty report spending up to 50 hours per month on simulation education.

EM has been on the forefront of innovative teaching solutions using sound andragogical theory. Without clear delineation of protected educational time for faculty, we will necessarily decrease educational innovation and effort in order to accommodate increased clinical expectations. This will degrade the educational experience for the residents and adversely affect patient safety and the clinical learning environment. The quality of the training environment impacts patient outcomes during training, and this effect remains stable after graduation.^7^, Without the explicit requirement of protected time for EM faculty to teach, this time will be lost due to the market forces described below. It is clear that the inability to train EM residents for rare, but high-risk clinical situations will have a profound negative impact on training, and will be transmitted to the public, as the population of inadequately-prepared residents grows.

We must also consider how the proposed rule changes will impact physician burnout. According to Medscape’s Annual National Burnout and Depression Report 2018, 8 EM has one of the highest burnout rates. A study published in *Archives of Internal Medicine* in 2012 reported that EM physicians were three times more likely to develop burnout than the average physician.^9^ The following factors have been identified as drivers of burnout and decreased engagement: workload/job demands, efficiency/resources, meaning in work, culture/values, control/flexibility, social support/community work, and work-life integration.^10^ The changes in support for faculty time in academic settings will have significant impact on the workload/job demands and meaning in work categories. Increasing ED volumes, charting demands, and emphasis on throughput metrics have negatively impacted the teaching environment.^11^ Faculty at institutions with residency programs consider it part of their mission to educate the next generation of EM physicians. If the balance of clinical service and education is shifted by increasing workload and decreasing time to educate, there will be a negative impact on faculty physician wellness and an increase in burnout.

Additionally, EM practice is becoming ever-more privatized and consolidated into large contracted medical groups (CMG). These corporations are large, for-profit companies that are incentivized to have their employees (EM physicians) see patients and generate revenue rather than spend time on educational or academic pursuits. This market pressure will begin to force CMGs that wish to remain lean and competitive to disincentivize academic and education time. This will absolutely and inevitably degrade the high standards to which EM educators hold their learners, and endanger patients both at those training sites and beyond.^7^

Results from a recent internal CORD survey queried Program Directors, Assistant Program Directors, and Core Faculty in US EM training programs. With almost 200 respondents, 95% reported that removal or decrease of core faculty protected time would be “job threatening” or “career threatening.” Likewise, over 96% of respondents reported that a loss of protected time would impede their ability to perform their academic duties to a large extent. Additionally, more than 99% of core faculty responding felt there would be a distinct negative impact from the loss of academic protected time.

With increasing volumes and charting demands, greater range of responsibilities, and no protected non-clinical time to teach outside of the clinical setting for Core Faculty, education outside of the clinical setting will be left solely to Program Directors and Associate/Assistant Program Directors without provisions for additional protected time for them. This will further erode resident education as well as Core Faculty and Program Director wellness. Additionally, it will have a negative impact on academic scholarship, when that is no longer seen as something worth time to cultivate.

Changes in requirements of dedicated non-clinical time for EM education faculty will lead to decreased scholarship, diminished exposure of residents to varied ways of thinking and practice, and a workforce of EPsthat are only incentivized by how fast patients can be moved through an ED. While external forces have decreased the amount of time available to teach during clinical shifts, removing protected time for Core Faculty to engage in education away from the bedside will diminish the amount and quality of that education. We need to protect our village to innovate and continue to advance EM education, creating the leaders of the future.
